# “Não tem vaga, só se conhecer alguém”: percepção do acesso à saúde por mulheres congolesas residentes no Rio de Janeiro, Brasil

**DOI:** 10.1590/0102-311XPT196524

**Published:** 2025-11-07

**Authors:** Paula Colodetti, Francisco Ortega

**Affiliations:** 1 Universidade do Estado do Rio de Janeiro, Rio de Janeiro, Brasil.; 2 Universidade Estácio de Sá, Rio de Janeiro, Brasil.; 3 Institució Catalana de Recerca i Estudis Avancats, Barcelona, España.; 4 Centre de Recerca en Antropologia Mèdica, Universitat Rovira i Virgili, Tarragona, España.

**Keywords:** Acesso a Serviços de Saúde, Mulheres, Refúgio, Racismo, Capital Social, Access to Health Services, Women, Shelter, Racism, Social Capital, Acceso a los Servicios de Salud, Mujeres, Refugio, Racismo, Capital Social

## Abstract

Dados sobre a saúde da mulher negra e sua presença como tema em periódicos nacionais ainda são escassos, principalmente se considerarmos o quão frequente é seu uso da rede de atenção primária e do Sistema Único de Saúde (SUS). Neste artigo, partimos de uma ótica interseccional sobre as categorias de gênero, raça, classe e nacionalidade, interligadas ao conceito de “capital social”, para analisar percepções dos serviços de saúde por mulheres congolesas residentes no Rio de Janeiro, Brasil, no ano de 2018. Trata-se de estudo qualitativo, composto por observação participante e oito entrevistas semiestruturadas, em uma instituição de acolhimento a refugiados, a Cáritas RJ. As narrativas apresentadas por essas mulheres expõem o impacto da violência cotidiana em suas experiências ao chegarem ao Brasil em busca de abrigo. Desconfiança, limitações com a língua e a falta de conhecimento contribuem para a carência de capital social e inacessibilidade dos serviços. Destacamos que as mulheres negras compõem um grupo grande de usuárias do SUS e, portanto, contar com sua participação ativa pode impactar suas experiências com esses serviços. Nesse sentido, ter espaços para a escuta de grupos vulneráveis, como o de congolesas refugiadas, é mais uma estratégia a fim de promover equidade e o acesso universal à saúde para todos.

## Abstract (en)

Data on the health of black women and the presence of this topic in national journals remain scarce, especially considering the frequent use of the primary care network and the Brazilian Unified National Health System (SUS, acronym in Portuguese) by these women. In this article, we take an intersectional perspective on the categories of gender, race, class, and nationality interlinked to the concept of “social capital” to analyze the perceptions of healthcare services by Congolese women residing in Rio de Janeiro, Brazil, in 2018. A qualitative study was conducted, consisting of participant observation and eight semi-structured interviews conducted at a refugee shelter (Cáritas RJ). The narratives of these women reveal the impact of daily violence on their experiences upon arriving in Brazil to seek shelter. Mistrust, language limitations, and lack of knowledge contribute to the lack of social capital and inaccessibility of services. As black women compose a large group of individuals that use the SUS, their active participation could exert an impact on their experiences with these services. The existence of spaces for listening to vulnerable groups, such as Congolese refugees, is another strategy for promoting equity and universal access to health for everyone.

## Abstract (es)

Los datos sobre la salud de las mujeres negras y su presencia como tema en las publicaciones periódicas nacionales siguen siendo escasos, sobre todo si tenemos en cuenta la frecuencia con la que recurren a la red de atención primaria y al Sistema Único de Salud (SUS). En este artículo, partimos de una perspectiva interseccional sobre las categorías de género, raza, clase y nacionalidad, interrelacionadas con el concepto de “capital social”, para analizar la percepción de los servicios de salud por parte de las mujeres congoleñas residentes en Río de Janeiro, Brasil, en el año 2018. Se trata de un estudio cualitativo, compuesto por observación participante y ocho entrevistas semiestructuradas, en una institución de acogida de refugiados (Cáritas RJ). Los relatos que cuentan estas mujeres muestran cómo la violencia diaria les afectó cuando llegaron a Brasil buscando refugio. La desconfianza, las limitaciones con el idioma y la falta de conocimientos contribuyen a la falta de capital social y a la inaccesibilidad de los servicios. Destacamos que las mujeres negras constituyen un grupo numeroso de usuarias del SUS y, por lo tanto, contar con su participación activa puede influir en sus experiencias con estos servicios. En este sentido, disponer de espacios para escuchar a los grupos vulnerables, como el de las refugiadas congoleñas, es otra estrategia más para promover la equidad y el acceso universal a la salud para todos.

## Introdução

A saúde passa a ser direito universal e dever do Estado no Brasil a partir da *Constituição Federal* de 1988 e é instituída pela *Lei nº 8.080/1990*, que regulamenta o Sistema Único de Saúde (SUS). No entanto, a efetivação da universalidade e integralidade esbarra desde seu início na insuficiência estatal em garanti-las, resultando num acesso desigual aos serviços ^1^. Nesse contexto, os grupos mais vulneráveis são prejudicados, mantendo-se fora dos círculos de funcionamento institucionais ou seus servidores.

O SUS conta ainda com um terceiro princípio fundamental, a equidade, que tem como proposta reconhecer demandas de grupos específicos a fim de reduzir o impacto dos determinantes sociais da saúde. Admitindo o desequilíbrio socioeconômico no país, e “*compreendendo a situação de iniquidade e vulnerabilidade que afeta a saúde da população negra*” ^2^ (p. 23-4), o Ministério da Saúde estabelece em 2009 a Política Nacional de Saúde Integral da População Negra (PNSIPN). Embora a cartilha não inclua a situação de migrantes e/ou refugiados é importante salientar que ao estudarmos um grupo de mulheres africanas, congolesas, e solicitantes de refúgio estamos nos referindo a pessoas duplamente vulnerabilizadas ^3^.

Ademais, há uma fragilidade na relação desse grupo com a população local ^3^, e, neste sentido, destacamos sua desvantagem, já que performam melhores índices de saúde as sociedades ou grupos sociais mais coesos socialmente. Diversos estudos analisaram a relação entre a participação das pessoas nas atividades coletivas e nos espaços públicos e a saúde dessas populações, o que foi atribuído ao capital social ^4^ ^,^ ^5^ ^,^ ^6^. O conceito de capital social ajuda a esclarecer como a união de pessoas em grupos permite a formação de organizações e gestos solidários recíprocos que fortalecem vínculos e promovem o cuidado consigo mesmos e com aqueles que fazem parte da rede ^5^ ^,^ ^6^, proporcionando bem-estar e melhores parâmetros em saúde para seus componentes.

Esse senso de unidade social ou entre grupos acaba por favorecer sentimentos de solidariedade, o que promove a resiliência e a formação de uma sociedade mais saudável, no sentido de que os participantes se sentem mais respaldados em suas demandas quando se reconhecem como membros de uma comunidade em que é possível receber apoio mútuo. Por outro lado, não fazer parte de uma associação acaba por causar o oposto, restringindo oportunidades e promovendo violência e distanciamento, seja no campo da saúde ou assistencial ^5^ ^,^ ^6^.

Santos & Farias Filho utilizam a noção de capital social para analisar as ações dos agentes comunitários de saúde (ACS) e refletir como as “*redes sociais formais e informais, estabelecidas pela confiança dos participantes,* [levam ao] *usufruto de vantagens geradas pelo conhecimento*” ^7^ (p. 1660). É nessa perspectiva que podemos entender falas como: “*só é atendido quem conhece alguém...*”, sinalizada por uma das mulheres congolesas solicitantes de refúgio, ouvidas no ano de 2018 pela principal autora deste artigo (P.C.), e reafirmada por todo esse grupo de mulheres, quando se encontravam reunidas numa “roda de conversa” realizada mensalmente à época, na Cáritas Arquidiocesana do Rio de Janeiro (Cáritas RJ).

Este artigo parte de falas dessas mulheres africanas, negras, periféricas e em situação de refúgio na cidade do Rio de Janeiro para situar o quanto a sensação de desconfiança aparece como barreira ao acesso às unidades de atendimento em saúde, diminuindo a procura pelos serviços e gerando descrédito quanto à possibilidade de serem abordadas com o devido acolhimento ou de terem benefícios dos tratamentos médicos.

Vale destacar que refugiados são migrantes forçados, pessoas que necessitaram evadir de seus países por motivo de ameaças a sua integridade, seja por causas naturais ou políticas, com grave violação de direitos humanos. No caso das mulheres abordadas nesta pesquisa, todas encontravam-se na condição de solicitantes de refúgio, quando já haviam feito o deslocamento, mas aguardavam o país receptor as reconhecerem enquanto tal ^8^.

São mulheres que chamam a atenção para a interposição entre a condição de migrante internacional - sem conhecimento da língua nativa ou das normas e direitos locais -, o racismo e a vulnerabilidade financeira, como fatores ligados ao descaso com que são recebidas nos serviços de saúde, provocando sua descrença nas instituições. Situação como a descrita por Horta et al. quando referem que seus interlocutores, mencionam “*demora no atendimento*” ^9^ (p. 113), falta de médicos, e a discriminação nos serviços de saúde - o que atribuíram ao fato de serem africanos - como fatores de afastamento e postergação a novas procuras.

Maia & Azize ^3^, por sua vez, destacam as disputas no território, e como essas se relacionam com aos atendimentos em saúde. Os ACS, que são moradores locais e usufruem dos mesmo serviços, transmitem a preocupação dos profissionais de saúde de que, ao oferecer a utilização dos recursos aos migrantes, poderiam faltar meios para a população local. Além disso, quando esses agentes registram os novos chegados e esses se deslocam do território, o que ocorre com frequência, o profissional fica suscetível a sofrer “*sanções burocráticas decorrentes de quaisquer quadros clínicos com desfechos negativos* [relacionados àqueles registrados e não mais encontrados]” ^3^ (p. 1793).

Em outro estudo, que avaliou a adaptação de congoleses no Rio de Janeiro, Ishizuka & Brulon ^10^ destacam as falas de seus entrevistados, neste caso todos homens, que uma vez atendidos nos serviços de saúde tiveram uma experiência semelhante à relatada pelas interlocutoras mencionadas neste artigo, ou seja, de negação de atendimento por supostamente não terem a “documentação necessária” ou de discriminação: seja por serem migrantes, seja por não falarem a língua nativa.

Neste contexto, examinamos, portanto, a influência do capital social, ou sua falta, nos arranjos dos atendimentos em saúde oferecidos a esse grupo composto por mulheres congolesas solicitantes de refúgio, sob a ótica interseccional, considerando assim todas as categorias de vulnerabilidade que afetam esse grupo. São situações que exigem que ultrapassemos a retórica da equidade para efetivamente podermos oferecer espaços de atendimento verdadeiramente inclusivos ^1^ ^,^ ^3^ ^,^ ^10^.

Nessa proposta, Santos & Farias Filho ^7^ apresentam a exploração do conceito de capital social em três níveis: o capital social de ligação (relações entre pessoas que compõem um mesmo grupo); de ponte (relação entre aqueles que compõem um grupo e sua relação com outro grupo da mesma categoria hierárquica); e de conexão (relação entre os componentes de um grupo e seus superiores hierárquicos). Neste artigo, refletimos sobre essa última categoria, tensionando o vínculo entre um grupo de mulheres congolesas e os dispositivos de saúde, procurando entender de que forma esse contato influencia o cotidiano dos serviços oferecidos pelo SUS.

A investigação acerca da troca entre pessoas em suas articulações rotineiras, considerando a proposta analítica do conceito de capital social, permite destrinchar lacunas na promoção em saúde que incidem no acesso dos usuários aos serviços e nas dificuldades e atitudes das equipes de saúde, que por sua vez afetam a maneira como esses arranjos repercutem no sucesso de um atendimento oferecido. Ainda, as especificidades do campo, como os desconhecimentos da língua, da rede assistencial e seus trabalhadores, e de seus direitos no Brasil erguem barreiras que não só postergam tratamentos, mas geram sentimentos de desconfiança nos serviços e profissionais de saúde.

As mulheres congolesas, por vezes sozinhas no Rio de Janeiro, se preocupam com a falta de recursos financeiros, a residência em áreas violentas e a discriminação encontrada no local. São preocupações que extrapolam o campo da saúde, mas que incidem diretamente sobre ele, seja pelo aumento dos níveis de estresse, seja por vivências de injustiça social que impactam na qualidade de vida e na saúde mental ^3^ ^,^ ^9^ ^,^ ^10^.

Utilizar o capital social para refletir sobre as experiências dessas mulheres congolesas refugiadas com as instituições brasileiras, nesse caso representado pelas Clínicas da Família e demais serviços de saúde oferecidos, nos permite compreender as barreiras de acesso aos serviços de saúde pelas populações vulneráveis.

## Metodologia

Foi realizado um estudo qualitativo, composto por observação participante e entrevistas, realizadas durante os meses de março e agosto de 2018, na Cáritas RJ - uma instituição católica de acolhimento à população refugiada. A Cáritas RJ é uma organização que surgiu a partir da iniciativa do bispo Dom Eugênio Sales, em 1976, no contexto de perseguição política dos períodos de ditadura, tanto no Brasil, quanto em países vizinhos. Desde então, presta assistência aos migrantes que chegam ao país em busca de acolhimento, e os orientam em relação a burocracias e direitos, entre eles, o direito à saúde ^8^.

O contato inicial com o campo se deu por meio de observação participante, o que ocorreu de forma exclusiva por dois meses. Neste período, a primeira autora (P.C.) deste artigo manteve presença semanal nas atividades de ioga e relaxamento oferecidas pela Cáritas RJ, onde frequentavam migrantes e solicitantes de refúgio/refugiados vindos de vários países. As aulas eram geralmente compostas por grupos de 20 participantes, entre homens e mulheres, e tinham duração de 50 minutos. A permanência nessa atividade pela pesquisadora ainda se estendeu por mais um mês, totalizando três meses - março a maio de 2018.

A intenção da pesquisadora ao participar das atividades era ser conhecida, o que se seguiu com a manutenção da frequência nas “rodas de conversa entre mulheres” (cinco encontros), e com o início das entrevistas. Estas foram realizadas no período de maio a agosto de 2018. As rodas de conversa eram temáticas, duravam cerca de uma hora e eram compostas exclusivamente por mulheres de várias nacionalidades, com presença da tradutora institucional - uma mulher congolesa refugiada e servidora da Cáritas RJ, contratada devido a superioridade numérica dessa população na época do trabalho de campo.

Durante essas rodas de conversas, as interlocutoras foram apresentadas à pesquisa, e solicitada a autorização de registrar suas falas por meio das notas de campo, o que foi aceito por todas elas. O convite à entrevista foi feito a mulheres congolesas participantes desses encontros, de forma individual, ao término dos mesmos. Os depoimentos trazidos a partir dos grupos são exclusivamente de mulheres congolesas e surgiram em um encontro sobre “acesso à saúde no território”. Vale destacar que antes de cada fala era pedido a elas que se apresentassem.

Foram realizadas oito entrevistas semiestruturadas em profundidade, gravadas em áudio e na presença de tradutoras: duas estudantes de psicologia que participavam à época de projetos na Cáritas RJ. Após a tradução simultânea, para transcrição, contou-se com a ajuda de uma dessas estudantes para o devido registro das falas. Das oito participantes, duas dispensaram as tradutoras por considerarem que já podiam se comunicar bem em português e por assim entenderem que seus depoimentos ficariam mais fielmente registrados.

As entrevistas e o material registrado nos encontros em grupo foram reunidos em categorias e universos de sentido. Os dados foram submetidos à verificação qualitativa, utilizando a análise de conteúdo proposta por Bardin ^11^.

A fim de preservar as identidades das participantes, foram-lhes atribuídos nomes diferentes dos seus próprios, porém, também de origem africana. Os Quadros 1 e 2 mostram esquematicamente alguns dados que se referem ao perfil sociodemográfico das entrevistadas ^12^.

O estudo foi aprovado pelo Comitê de Ética e Pesquisa do Instituto de Medicina Social da Universidade do Estado do Rio de Janeiro (CEP-IMS/UERJ; parecer nº 2.503.739).

**Quadro 1:** Perfil sociodemográfico das participantes: residência, escolaridade e empregabilidade.

NOME	IDADE (ANOS)	CHEGADA AO BRASIL	BAIRRO ONDE RESIDE NO RIO DE JANEIRO	ESTÁ EMPREGADA *	ESCOLARIDADE **
Aisha	47	2012	Penha	Sim	Superior completo
Badu	27	2017	Barros Filho	Não	Escolarizada
Chinu	30	2017	Barros Filho ***	Sim ^#^	Escolarizada
Halima	60	2014	Penha	Não	Escolarizada
Jani	27	2017	Curicica	Não	Escolarizada
Kalifa	29	2015	Brás de Pina	Sim	Superior incompleto
Nia	20	2017	Estácio	Sim	Superior incompleto
Oni	27	2017	Barros Filho ***	Sim ^#^	Escolarizada

* Nenhuma das participantes, mesmo aquelas que trabalham regularmente, está atuando em sua área de formação;

** Como foi difícil o entendimento sobre o grau de escolaridade, registrou-se “Escolarizada” para quem declarou ter frequentado a escola, englobando Ensino Fundamental e Médio, em todos os casos, incompleto. Para quem cursou Ensino Superior, foi especificado se completo ou incompleto;

*** As participantes Chinu e Oni residem juntas;

^#^ Sem vínculo empregatício formal.

**Quadro 2:** Perfil sociodemográfico das participantes: estado civil e dependentes.

NOME	ESTADO CONJUGAL	RESIDE COM O PARCEIRO	NACIONALIDADE DO PARCEIRO	NÚMERO DE FILHOS	FILHOS QUE RESIDEM CONSIGO
Aisha	Casada	Não	República Democrática do Congo	3	1
Badu	Separada *	Não	República Democrática do Congo	3	2
Chinu	Solteira	Não	Não se aplica	0	0
Halima	Viúva	Não	República Democrática do Congo	7	0
Jani	Solteira	Não	Não se aplica	0	0
Kalifa	Casada	Sim	República Democrática do Congo	1	1
Nia	Solteira	Não	Não se aplica	0	0
Oni	Solteira	Não **	República Democrática do Congo	0	0

* Seu estado civil legal é casada, porém, considera-se separada;

** Tem namorado natural da República Democrática do Congo, também refugiado e residente em São Paulo.

## Resultados e discussão

No ano de 2018, em que foi realizado o trabalho de campo para esta pesquisa, houve a intervenção militar no Município do Rio de Janeiro. Tratava-se de um período de exceção, marcado por ações estatais autoritárias e violentas, e por uma colaboração entre polícias e forças armadas. Situações, que, segundo as participantes, traziam um constante sentimento de tensão e vulnerabilidade, que não esperavam encontrar ao chegarem no novo território, mas que as atingiu diretamente em suas rotinas - em particular, por serem mulheres negras estrangeiras, com poucos recursos financeiros e residentes em zonas periféricas.

No caso da saúde, em especial, estratégias pensadas para serem universais, por vezes, falham ao tentarem atingir grupos específicos. Além disso, é necessário que tais estratégias também considerem o recebimento dos usuários nas unidades, já que diversos estudos mostram a interferência da racialização nos serviços oferecidos, seja na maneira como as pessoas são recebidas, em como os atendimentos são executados, ou outros aspectos que dizem respeito às condutas da equipe de saúde ^3^ ^,^ ^13^ ^,^ ^14^ ^,^ ^15^ ^,^ ^16^.

Neste contexto, mesmo com o estabelecimento das PNSIPN ^2^, que tratam dos atendimentos às pessoas negras, ou com o estabelecimento do Plano Estadual de Políticas de Atenção aos Refugiados, implementado no Rio de Janeiro em 2014 ^10^, encontramos no território disputas ^3^ que representam uma barreira no acesso aos serviços de saúde para os refugiados, que se traduz na resistência das equipes em recebê-los, nas dificuldades de comunicação, ou por não possuírem a documentação necessária para o atendimento ^3^ ^,^ ^9^ ^,^ ^10^ ^,^ ^12^ ^,^ ^13^ ^,^ ^14^.

Quando não refletimos sob a ótica interseccional sobre as desigualdades a que estão expostas as mulheres negras periféricas, às quais neste estudo soma-se a categoria de refúgio, contribuímos para a invisibilização de uma série de fatores de vulnerabilidade a que estão expostas. Ora, se o estresse e o acometimento psíquico afetam diretamente a qualidade de vida e a saúde dos indivíduos, a distribuição de renda e do capital social tem um impacto na saúde, já que “*a extensão da desigualdade material é um dos principais determinantes do bem-estar psicossocial nas sociedades modernas e o impacto na saúde é apenas um dos custos sociais que acarreta*” ^4^ (p. 9; tradução livre).

Como denuncia Lélia Gonzalez ^17^ (p. 111), a “*mulher, negra e pobre* [vive] *acuada à espreita do próximo golpe a ser recebido, vigiando-se e ‘saindo de cena’ para não ser mais ferida do que já é*”. Essa sensação de desconfiança as acompanha desde o contato com o colonizador, seja no Brasil ou na África ^18^, e permeia as narrativas e percepções das mulheres aqui mencionadas. Essa desconfiança constitui neste estudo uma barreira importante de acesso aos serviços de saúde.

Nas narrativas a seguir, estão assinaladas com (P) as participantes das rodas de conversa que optaram por não ceder entrevistas individuais, mas estiveram de acordo com o registro de suas falas durante as atividades em grupo. Boa parte das nossas informantes questionava as longas filas de espera para receber atendimento ou a demora para consulta com um especialista, “*a não ser que conheçam alguém*”, dentro da unidade.

“*Consegui um exame porque uma senhora na fila - negra também - me viu esperando e perguntou o que eu queria. Quando soube do meu caso, disse que ia me ajudar porque conhecia alguém ali. Aí marcaram meus exames* [mamografia e ultrassonografia]*. Mas isso não está certo! Como faz se você não conhece ninguém?!*” (Kalifa).

Essa mesma mulher nos fala de outra vivência em que, assim como os interlocutores de Horta et al. ^9^, se queixa da demora nos atendimentos, dos entraves causados pelas dificuldades no entendimento entre servidores e paciente:

“*Com meu filho, esperei bastante pelo médico. Quando cheguei para falar com ele, tentei dizer, que estava com febre, abatido, mas ele nem olhou e nem esperou. Fez um remédio para dor e pronto. Não dá para ir num lugar assim. Se vai na clínica da família, tem que esperar, ‘não tem vaga’*” (Kalifa).

Como mencionado em diversos estudos, destacam-se depoimentos dos profissionais de saúde em relação ao desconforto de inserir migrantes e refugiados entre o rol de pacientes a serem assistidos ^3^ ^,^ ^9^ ^,^ ^10^ ^,^ ^12^ ^,^ ^13^ ^,^ ^19^ ^,^ ^20^, seja pelas dificuldades da língua, a sensação de que não entendem o funcionamento do sistema nem os tratamentos recomendados, a falta de documentação ou a necessidade de dividir com eles recursos já escassos. Estudar essas conexões é também uma forma de superá-las.

A gestão da vida determina não só os planos e expectativas futuras de uma população ou grupo populacional, mas influencia diretamente em seus níveis de saúde, em especial de saúde mental e no gerenciamento do estresse ^15^. Como várias revisões históricas demonstram, diferentes condições sociais repercutem diretamente no estado de saúde das pessoas ^4^ ^,^ ^14^ ^,^ ^15^ ^,^ ^16^, e a avaliação por meio dessa articulação com o capital social não perde de vista a noção do psiquismo, nem tampouco a de sobrecarga como importantes influências para piora dos quadros de saúde de uma sociedade.

Ainda, ao analisarmos a coesão social, incluindo a capacidade dos sujeitos de confiar em seus pares, instituições e burocracias estatais, verificamos a possibilidade de essas pessoas se manterem otimistas, de participarem de ações em comunidade, de estabelecerem vínculos e de esperarem que essas atitudes sejam recíprocas, o que influencia a esperança e a resiliência, sentimentos importantes para prevenção do adoecimento mental ^5^ ^,^ ^20^.

São relações dinâmicas, que ora se mostram mais harmônicas, ora mais tensas, como nos revela uma das entrevistadas:

“*Há dois tipos de brasileiros: há aqueles que são bons, que querem olhar para você e falar com você, te ajudar, mas há os que nem querem te ver, que não querem te encostar! Para fazer exame* [saúde, laboratoriais]*, aqui não tem jeito. Não tem vaga. O atendimento demora demais. Já desisti*” (Aisha).

As narrativas das nossas entrevistadas revelam como raça e nacionalidade interferem na percepção dessas mulheres sobre os serviços de saúde. Elas descrevem a sensação de menor duração dos atendimentos, barreiras na comunicação, maior tempo em filas para marcação de consultas e exames - o que associam ao fato de não conhecerem as pessoas que trabalham no local:

“[O atendimento para meu filho em uma UPA] *é sempre rápido.* [Mas] *parece que eles não ouvem, não querem ouvir, não têm paciência para escutar por conta da língua... Você aponta e eles dizem: tá, já sei onde está com dor, vou te dar o remédio. Nem fazem a receita direito... Lá tem racismo também! Eu como negra espero mais na fila. Vejo uma branca chegar e eles* [funcionários] *vêm, dão dois beijinhos e pronto, vai ser atendida! Eu fico lá e espero!*” (Kalifa).

As barreiras linguísticas se somam ao racismo e ao desconhecimento institucional nos comentários dessas refugiadas. Destacam-se a desconfiança e o baixo capital social:

“*Eu fui levar meu filho e o médico não quis esperar. Rapidamente olhou o ouvido, disse que o problema era no ouvido e deu o remédio. Tinha outra coisa que ele falou para fazer, mas não entendi direito. Ele falou, mas não deu receita. Pedi na farmácia e eles me ajudaram. Consegui comprar o antibiótico. Mas isso de marcar não consigo. Falam também que não tem vaga!*” (P).

A falta de confiança se associa diretamente ao aumento da violência territorial e é mais elevada nos lares liderados por mulheres ^4^ ^,^ ^21^ ^,^ ^22^. Das oito mulheres entrevistadas, apenas uma delas estava no Brasil com o marido e os filhos. Todas as outras tiveram suas famílias separadas no processo de refúgio.

São mulheres que relatam sensações frequentes de suspeição, seja em relação a seus pares, ou seja aos locais que frequentam. Esses sentimentos são recorrentes nos depoimentos das participantes da pesquisa, como narra, durante uma roda de conversa, uma de nossas entrevistadas, cuja profissão é trançar cabelos num estabelecimento de propriedade de outra mulher natural da República Democrática do Congo:

“*Há brasileiros que vêm e que dão dois beijinhos, outros que te olham com desconfiança. Tem racismo no Brasil sim. Minha filha está com umas coisas na pele e couro cabeludo* [micose]*. Levei na clínica da família e disseram: ‘ela não está bem, precisa tratar isso’. Aí perguntei se não tratariam ela e me falaram: ‘só depois que marcar com o pediatra para fazer o primeiro atendimento’. Então marca para ela! E elas: ‘só daqui a seis meses’. Aí não adianta. Ela está mal agora. O cabelo dela está caindo, está horrível*” (P).

O depoimento acima demonstra a dificuldade de entendimento do funcionamento dos fluxogramas do SUS, assim como a falta da capacidade de comunicação sobre esse funcionamento, algo que também acontece com os próprios brasileiros que frequentam as unidades de atendimento. Como apontam os estudos mencionados, esse desconhecimento, e a divergência de línguas e as disputas no território, colaboram para a menor disponibilidade dos servidores em atendê-los ^3^ ^,^ ^9^ ^,^ ^10^ ^,^ ^20^ ^,^ ^23^ ^,^ ^24^.

O sistema congolês, segundo as falas das próprias mulheres, funciona com pagamentos feitos pelos usuários após as consultas realizadas. Os casos de emergência ou mais graves são prontamente absorvidos pelo Estado. Portanto, o funcionamento do sistema brasileiro de saúde não lhes é familiar, o que traz questionamentos por parte dessas usuárias e por parte da equipe, uma contrarresposta negativa, que as faz evitá-las ^3^.

As indagações dessas mulheres durante as atividades em grupo ou nas entrevistas em relação às unidades de saúde ou aos serviços assistenciais como um todo são uma forma de contestarem uma rotina de opressão e ceticismo presente em seu cotidiano. São falas que trazem as diversas formas de violação inscritas sobre seus corpos, que se vinculam a suas histórias de vida ^21^ ^,^ ^22^ e que não cessam com a chegada no novo território. Seus questionamentos denunciam acontecimentos com que se deparam no território carioca e sobre a própria instituição que lhes é referência, a Cáritas RJ:

“*Vocês falam que a gente tem que falar, que tem que vir aqui e tem que falar. Mas não vejo mudar nada. Já venho aqui tem uns dois anos e não muda. Não sei para que fazer esse trabalho*” (Kalifa).

Se as marcas deixadas pelas recorrentes violências e o sentimento de invisibilidade estão presentes nessa espécie de absorção mútua do violento e do comum, algo que se faz presente em conjunto e silenciosamente: a vivência e a necessidade de expor o que acontece se perdem na falta de expectativa de que mudanças ocorram. Em conformidade com a violência cotidiana, as vozes se emudecem, “*não no sentido de que não se tem palavras, mas que essas palavras se tornam congeladas, dormentes, sem vida*” ^22^ (p. 8; tradução livre).

Na sensação rotineira de ter direitos violados, que se faz presente para além do sentido físico da violência, o medo e o ceticismo passam a ser incorporados dentro de cotidianos desgastados. Sem as garantias de pertencimento, situadas às margens das entidades maiores, tais como comunidades ou o Estado, essas mulheres demonstram sentimentos de traição ou abandono sobre os quais não vislumbram reparos ^21^ ^,^ ^22^.

Se a concepção de capital social busca estabelecer o nexo entre o impacto do sentimento de coletividade nas expectativas sobre o futuro, minimizando a sensação de desamparo e trazendo benefícios à saúde, ao trabalharmos esse conceito, desenvolvemos reflexões sobre as vulnerabilidades dos grupos minoritários e o quanto essa falta de rede interfere nos processos de saúde/doença ^4^ ^,^ ^6^.

A atenção primária à saúde (APS) pode desempenhar papel de destaque como “*agente transformador do racismo estrutural presente na sociedade e do racismo institucional na saúde.* [Por ser a APS] *o primeiro contato preferencial da população com o sistema de saúde, dialoga diretamente com a garantia (ou não) de acesso da população negra aos serviços*” ^25^ (p. 12). Nesse sentido, a existência de experiências de atendimento bem-sucedidas permite a construção de vínculos de confiança, trazendo esperança e influindo positivamente na saúde e na qualidade de vida dessas pessoas.

“*Eu tive uma experiência boa: minha filha estava passando mal. Um cara me viu e viu meu marido na rua. Aí ele parou o carro e levou a gente até a UPA. Lá foram bons com a gente. Como saí de casa com pressa, estava sem documentos direito, sem nada. Minha filha vomitou e se sujou toda. Uma outra moça me deu uma fralda e uma roupa da filha dela. Não tive problemas com isso aqui*” (P).

Para além das críticas ou elogios ao sistema de saúde durante as reuniões de grupo, a questão racial e as vivências discriminatórias ocuparam lugar de destaque nas entrevistas, nas quais demonstraram perplexidade ao se deparar com determinadas situações, como preconceito enfrentado pelos filhos nas escolas ou desentendimento entre pares, inclusive entre mulheres negras e pardas brasileiras.

“*Veio uma moça e me chamou de macaca. Ela era morena! Aí falei para ela que, se sou macaca, ela era macaquinha. Porque eu era a mãe dela. Foi isso que falei para ela: sua mãe deve ser igual a mim, porque assim são nossos antepassados...*” (Kalifa).

Além das observações sobre experiências discriminatórias cotidianas, nossas entrevistadas relataram a falta de oportunidade para negros no Brasil, a maior resistência para entrada de formados e especializados em suas áreas de trabalho, além de um olhar objetificado sobre o corpo da mulher negra, minando a confiança de terem melhores expectativas de futuro.

“*Graças a Deus não tenho nada* [em termos de doença]*. Com minha filha* [de 15 anos]*, tive sorte. Ela gosta de estudar. Ela até que fez muitas colegas, mas não deixo ela sair, nem namorar. No Congo, ela não sairia. Ela já até sabe. ‘Minha mãe não vai deixar’, ela fala... Me deram a oportunidade de trabalhar como arrumadeira, é o que faço no hotel agora. Sou formada, tenho universidade, mas aprendi que tenho que ficar com a oportunidade. Aceitar do jeito que vier...*” (Aisha).

A mesma entrevistada observa, em relação ao negro e à cultura brasileira:

“*Eu mesma não gosto de samba... Olho aquelas mulheres, são sempre negras, né? Quase nuas sambando... É aquilo que querem, mas o que fazem depois dali? Esse é o futuro. Tenho raiva do carnaval! Só as pretas sambam, os brancos se formam. É assim nas formaturas! Não tem nenhum negro! Eu via quando trabalhava no* [casa de shows onde se fazem eventos de formaturas]*, os negros estavam servindo! Quero minha filha estudando!*” (Aisha).

Como podemos notar, há entre elas vozes mais eloquentes e reativas, e outras marcadas pelo esgotamento. Mas, mesmo diante da vivacidade dos discursos, muitas vezes as maneiras que encontram para lidar com essas violências são frequentemente silenciosas:

“*Tive uma vizinha que me acusou de não ter botado o lixo na lixeira* [que fica a alguns metros de seu prédio]*, um lixo muito fedorento! A vizinha cismou que tinha sido eu, ‘porque eu tenho criança’.* [Atribuí a ideia de racismo à acusação] *tive medo da vizinha falar isso na ‘boca’ e eu ser repreendida. Mesmo assim, desci com aquele lixo... Com muita raiva, mas desci*” (Badu).

Diante desse tipo de narrativa, é possível perceber que as disputas entre vizinhos e a frágil relação de rede que aparece nos cotidianos é silenciada pela falta de alianças e a apreensão de que algo pior possa lhes ocorrer. Frente à insegurança, que se reforça ao estarem sozinhas no território, os silenciamentos não são necessariamente passivos e nem carentes de resistência.

A essa série de pontos de vulnerabilidade soma-se a presença da violência no território carioca, a maneira como são elas mesmas e como veem outras pessoas sendo abordadaos pelos agentes estatais e as diferenças sentidas em relação a essas práticas abusivas, mas rotineiras, em comparação com situações vividas em seu país de origem:

“*O Rio é violento. Da onde eu vim também é violento, mas aqui não sei se posso dizer que é mais violento... Não sei onde é mais violento. Mas aqui eu vejo que tem mais morte, mais gente inocente, vítima, morrendo. Eu evito andar de noite e ando sempre com oração... A diferença é que lá tem guerra e aqui não... Aqui tem mais assaltos e lá não tem muitos assaltos não* [morava na capital, Kinshasa]*. Aqui até os meninos têm arma, onde eu moro você pode ver um menino armado até com fuzil!*” (Badu).

Diante a falta de acesso ao mercado de trabalho formal, migrantes ^26^ e refugiados ^3^ ^,^ ^8^ ^,^ ^9^ ^,^ ^10^ muitas vezes irão residir em territórios periféricos como as favelas, termo utilizado para nos referirmos a essas comunidades situadas nas periferias das cidades, densamente povoadas de forma informal, marcadas pela presença do poder paralelo - grupos civis armados e envolvidos com atividades criminosas -, onde o Estado se faz ausente. Um lugar marcado pelo frágil equilíbrio da violência, em que decisões políticas podem desarticular ainda mais a tensão e os riscos diários. Nosso trabalho de campo coincide com um desses momentos, a intervenção militar no Rio de Janeiro, que pôs em suspensão os direitos civis, em nome de práticas estatais de exceção - “em nome da paz”. Práticas exercidas fora dos parâmetros constitucionais e dentro de uma lógica necropolítica - “*expressão máxima da soberania* [a estatal, que ditará]*, quem pode viver e quem deve morrer*” ^27^ (p. 5).

“*Vivemos na favela, porque a casa lá é mais barata... Lá tem tiroteios quase todos os dias, mas temos que suportar isso... É o lugar que podemos pagar. Lá na favela matam as pessoas na nossa frente... No Congo, havia uma guerra, mas a gente não via as pessoas atirarem assim umas nas outras, em todos os lugares. No lugar onde moro, tenho medo de falar com os brasileiros... Sei que tem gente bem-intencionada, mas tenho medo de falar com eles*” (Chinu).

Nessa perspectiva, mantêm-se excluídas as populações marginais e justificam-se atos de brutalidade “em nome dos interesses da nação” ^27^. Não menos hostis são os atos praticados contra facções rivais dentro desse território. A questão que surge então é que refúgios existem para negros, favelados, africanos, mulheres e todas suas intersecções. Enquanto contextos de guerra, Rio de Janeiro e República Democrática do Congo não são tão diferentes ^12^ ^,^ ^28^.

Para Farmer et al. ^29^, racismo, desigualdade de gênero, pobreza, violência política e guerra são fatores determinantes para estabelecer “quem adoece e quem tem acesso a cuidados”. Nesse sentido, os depoimentos dessas mulheres apontam para a possibilidade de olhar criticamente para esse sistema que garante privilégios. A falta de justiça social, como apontam Smolen & Araújo ^30^, se associa à maior ocorrência de transtornos mentais em raças não brancas (pardos e negros), assim como a suas vivências de vulnerabilidade e insegurança cotidianas.

Essas percepções, somadas à falta de conhecimento ou vínculos interpessoais estabelecidos, mobilizam sentimentos de desamparo, diante dos quais a igreja católica ou os cultos protestantes, com pastores nativos da República Democrática do Congo, presentes nesse território são seus principais pontos de apoio ^3^ ^,^ ^28^ ^,^ ^31^. São locais principalmente intracomunitários, em que o culto acontece na língua nativa da República Democrática do Congo, Lingala, fortalecendo as redes entre o grupo, porém, mantendo-os afastados das conexões com o território carioca ^3^ ^,^ ^10^ ^,^ ^12^. Por outro lado, os serviços assistenciais aparecem, em suas falas, como lugares de não compreensão, inacessíveis.

Como indicam Souza & Grundy ^5^ (p. 1358), existem benefícios e malefícios que podem ser atribuídos ao capital social. Entre os benefícios, podemos sublinhar “*a provisão de suporte mútuo e todas as vantagens derivadas das organizações sociais que os membros podem obter*”. Contudo, e pelos mesmos motivos, aqueles considerados “não associados”, podem ter suas oportunidades reduzidas em prol dos associados.

## Conclusões

Apesar de sugerirem a necessidade de uma definição mais precisa dos critérios envolvidos no conceito de capital social para seu uso em pesquisas no campo da saúde, Macinko & Starfield ^6^ assinalam que a falta de formação de redes e vínculos de confiança entre pessoas, e entre pessoas e instituições, reforçam inequidades sociais e inseguranças que têm a saúde mental como ponto de impacto central.

Promover o investimento em grupos populacionais pode ser fator motivador para que participem de ações preventivas e mobilizem os demais ^5^; por outro lado, é possível que aqueles que não se sentem parte de uma comunidade deixem de acreditar que possam ter acesso aos serviços ^3^ ^,^ ^9^, tal como nos mostrou a experiência dessas congolesas refugiadas, residentes no Rio de Janeiro.

Soma-se à percepção de não pertencimento o racismo observado por elas, o que, até então, era uma experiência de certa forma desconhecida, já que elas não vivenciaram a diáspora. Ao se defrontarem com tais aspectos, que vão desde a desvalorização de sua estética até a de suas potencialidades acadêmicas, isso influencia a forma como veem a si próprias, desacreditando em melhores possibilidades para si e seus familiares nesse território, além de afetar sua saúde. Privilegiar o cuidado às mulheres negras refugiadas, observando suas particularidades e promovendo ações afirmativas que estimulem sua participação na elaboração de prioridades assistenciais, faz-se central para que se promova uma atenção restauradora.

## Data Availability

Os dados de pesquisa estão disponíveis mediante solicitação à autora de correspondência.
